# The Herbicide Atrazine Activates Endocrine Gene Networks via Non-Steroidal NR5A Nuclear Receptors in Fish and Mammalian Cells

**DOI:** 10.1371/journal.pone.0002117

**Published:** 2008-05-07

**Authors:** Miyuki Suzawa, Holly A. Ingraham

**Affiliations:** 1 Department of Cellular and Molecular Pharmacology, University of California San Francisco, San Francisco, California, United States of America; 2 Department of Physiology, University of California San Francisco, San Francisco, California, United States of America; Smithsonian Institution, United States of America

## Abstract

Atrazine (ATR) remains a widely used broadleaf herbicide in the United States despite the fact that this s-chlorotriazine has been linked to reproductive abnormalities in fish and amphibians. Here, using zebrafish we report that environmentally relevant ATR concentrations elevated zcyp19a1 expression encoding aromatase (2.2 µg/L), and increased the ratio of female to male fish (22 µg/L). ATR selectively increased zcyp19a1, a known gene target of the nuclear receptor SF-1 (NR5A1), whereas zcyp19a2, which is estrogen responsive, remained unchanged. Remarkably, in mammalian cells ATR functions in a cell-specific manner to upregulate SF-1 targets and other genes critical for steroid synthesis and reproduction, including Cyp19A1, StAR, Cyp11A1, hCG, FSTL3, LHß, INHα, αGSU, and 11ß-HSD2. Our data appear to eliminate the possibility that ATR directly affects SF-1 DNA- or ligand-binding. Instead, we suggest that the stimulatory effects of ATR on the NR5A receptor subfamily (SF-1, LRH-1, and zff1d) are likely mediated by receptor phosphorylation, amplification of cAMP and PI3K signaling, and possibly an increase in the cAMP-responsive cellular kinase SGK-1, which is known to be upregulated in infertile women. Taken together, we propose that this pervasive and persistent environmental chemical alters hormone networks via convergence of NR5A activity and cAMP signaling, to potentially disrupt normal endocrine development and function in lower and higher vertebrates.

## Introduction

Endocrine disrupting chemicals (EDCs) affect the reproductive health of fish and amphibious wild life [1], but their impact on mammals and particularly humans is less clear. Synthetic and natural endocrine disruptors fall into several chemical categories and include industrial chemicals, pesticides and herbicides. Some of these EDCs, such as the active chemical found in polycarbonate containers, bisphenol A, exhibit estrogenic effects in cultured cells [2], [3], [4], [5], by binding directly to the estrogen receptors ERα and ERβ [6], [7], [8]. However, other EDCs fail to competitively bind ERs, including the widely used chlorotriazine herbicide atrazine (ATR) [8], [9].

The prevalent use of ATR as a broadleaf herbicide and its persistence in the environment underscores the importance of understanding the molecular impact of this EDC. Numerous studies in fish, amphibians, reptiles and mammals all suggest that ATR can alter normal endocrine, neuroendocrine and immune responses. For instance, in amphibians, low levels (0.1–25 µg/L) or short term exposure (48 hrs) to ATR, respectively, increases the number of intersex frogs [10], [11], [12], [13], and impairs normal gonadal development [14], [15]. Consistent with these phenotypes, acute exposure to ATR lowers testosterone levels and impairs gonadal development in young fish [16], [17], in the developing alligator [18], and in young peripubertal male rats [19]. However, other studies suggest that reduced serum testosterone after ATR exposure results from a marked drop in body weight and food consumption. These latter effects are observed for both male and females rats and potentially reflect an unknown role of ATR in neuroendocrine signaling [20], [21], [22]. Still others suggest that independent of body weight and hormone levels, ATR delays mammary gland development [23]. Although there is ample literature documenting the effects of ATR in a variety of species, with the exception of aromatase (Cyp19A), other molecular targets of ATR remain poorly defined.

**Figure 1:**
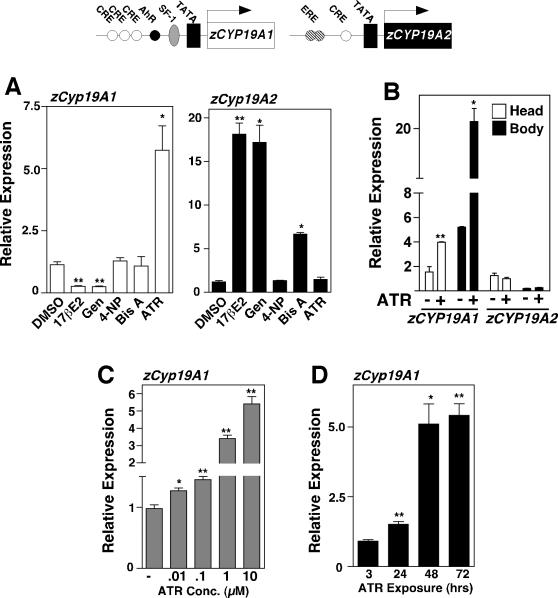
ATR stimulates expression of gonadal zcyp19A1 encoding aromatase, but not zcypA2 in zebrafish. Schematic of zCyp19a1 and zCyp19a2 zebrafish promoters with binding sites and start site indicated (arrow) as previously shown by [29]. A. Relative expression of zCyp19a1 and zCyp19a2 transcripts determined by RT-qPCR in juvenile zebrafish (20 dpf) following exposure (72 hrs) to endocrine disruptors including 0.1 µM of 17βE2, 1 µM of genistein (Gen), 10 µM of 4-nonylphenol (4-NP), 10 µM of bisphenol A (Bis A) and 10 µM of atrazine (ATR) beginning at 17dpf. B. Relative expression of zCyp19a1 and zCypA2 transcripts in dissected 20 dpf zebrafish bodies and heads after 72 hrs ATR treatment (10 µM). C. Relative zCyp19a1 and zCyp19a2 transcript levels are shown with ATR stimulation at doses ranging from 0.01 to 10 µM for 72 hrs (left panel), or at different time points (hrs) with 10 µM ATR (right panel). For all panels error bars represent the S.E.M. obtained from analysis of three independent groups of fish (n = 5) using validated primers, with reactions carried out three times each. T-test analysis reveal statistical significance with **p<0.01, *p<0.05.

Maintenance of cytochrome p450 aromatase activity, which catalyzes the conversion of androgens to estrogens appears critical for preserving a balanced sex ratio in teleosts. All species lacking sex chromosomes, such as fish [24], are especially sensitive to environmental factors that perturb sex steroid levels. Indeed, increasing estrogen levels in a natural or laboratory setting feminized [25], and greatly altered normal sex ratios in fish [26]. Conversely, treatment with aromatase inhibitors results in gonadal masculinization of female fish [27], [28]. This fact positions aromatase as a potentially useful target to examine the in vivo effects of ATR. In mammals, both ERα and the NR5A nuclear receptor, steroidogenic factor 1 (SF-1) influence expression of the single Cyp19A gene encoding aromatase. In zebrafish (*Danio rerio*), regulating aromatase expression is more complex because of gene duplication. The gonadal-enriched zcyp19a1 contains an NR5A binding site and is presumably activated by the zebrafish SF-1/LRH-1 orthologs (ff1a, b, c and d) [24], whereas the brain-enriched zcyp19a2 contains an estrogen response element (ERE) and is responsive to estrogens [29], [30] (Fig 1). Both zebrafish aromatase promoters contain CREB binding sites and would therefore be responsive to cAMP signaling. Not surprisingly, studies using several fish species showed upregulation of zcyp19a2, with a notable downregulation of zcyp19a1 after exposure to estrogen, xenoestrogens and other estrogenic chemicals [31], [32]. While a direct link between ATR and zcyp19a1 has not be established, others have shown that at relatively low doses (0.1 µM or 22 µg/L) ATR greatly increases aromatase activity in selective mammalian cell lines [33] and in immortalized sea turtle cells [34].

Recently, ATR has been proposed to bind and activate SF-1 [35]. This notion is particularly appealing given that SF-1 orthologs are found in all vertebrates including teleosts, and given the critical role of SF-1 in mammalian sexual development and steroidogenesis [36], [37]. Here, we used mammalian cell lines and zebrafish as model systems to address the in vivo and in vitro roles of ATR in activating aromatase expression. We specifically asked whether Cyp19A1, a known target of the NR5A receptors (SF-1, LRH-1, ff1d), would be selectively activated by ATR using these different model systems. Based on our collective data, we hypothesize that all NR5A receptors and tissues expressing these receptors are especially sensitive to the effects of ATR. Additionally, we propose that at environmentally relevant concentrations this herbicide does indeed act as an endocrine disruptor in fish, and based on our cellular data has the potential to influence the developing and adult endocrine system in mammals.

## Results

### ATR upregulates zcyp19a1 and alters the sex-ratio in zebrafish

To determine whether ATR and other endocrine disruptors might affect NR5A receptor activity in vivo, sexually immature zebrafish larvae were exposed to different chemicals for their potential to regulate zcyp19a1. Using quantitative PCR (qPCR) we found robust increases in expression of zcyp19a1, but not zcyp19a2 after acute exposure to ATR (1 µM) in 17 days post fertilization (dpf) zebrafish (Fig 1A). As shown previously [31], estradiol (E2), the phyto-estrogen, genistein, and the industrial chemical bisphenol A, all elevated the relative expression of zcyp19a2 after 3 days of exposure consistent with an ERE present in the proximal promoter (Fig. 1A). The induction of zcyp19a2 by estrogens (E2, genistein) is in stark contrast to their significant down regulation of zcyp19a1 (Fig 1A, left panel). Further analysis of these two aromatase transcripts in the zebrafish head and body confirmed that ATR exposure only affected zcyp19a1, but had no effect on expression of the brain-enriched zcyp19a2 (Fig 1B). Regulation of zcyp19a1 by ATR was dose and time dependent with maximal effects detected at 48 hrs, and with 10 µM of drug added (Fig 1C, D). Statistically significant responses were observed at lower doses of 0.01 and 0.1 µM ATR, which equate to 2.2 to 22 ppb (µg/L), respectively. Reported ATR concentrations in impacted agricultural areas are reported to be 6 to 20 ppb [11], illustrating that significant increases in endogenous aromatase expression are observed at ecologically relevant levels of this compound.

**Figure 2:**
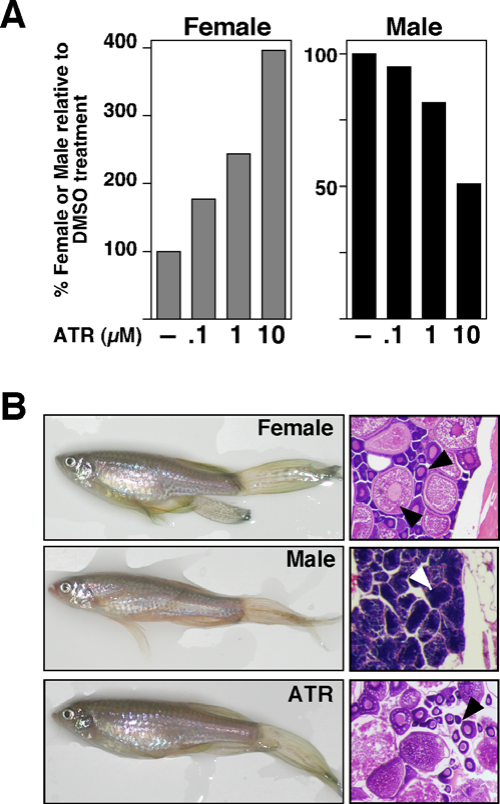
Chronic exposure to ATR increases the percentage of female zebrafish. A. The percentage of female zebrafish is shown after chronic exposure to DMSO and increasing ATR concentrations (µM). The number of female fish was assessed visually at three, four (data not shown) and at six months, as shown. Visual inspection for sex-specific landmarks (body shape, body color, fin shape) at both stages suggested an increase in the ratio of female to male fish. Following visual inspection of six month old fish, unambiguous assignment of sex was determined by morphological inspection of gonads. ATR treatment began at 17dpf post-hatching. B. Representative pictures of fish treated with ATR and corresponding gonadal sections stained with hematoxylin and eosin. Oocytes within the perinuclear follicles of the ovary are indicated (black arrowhead), and spermatozoa in the testis are indicated (white arrowhead).

Given that ATR increased endogenous levels of gonadal zcyp19a1 after short exposures, we asked if the ratio of male to female fish would be influenced by chronic exposure (six months) to this herbicide. Indeed, a dose-dependent increase in the number of female fish was observed with a corresponding drop in the male fish after ATR exposure when compared to the control tank with DMSO (Fig 2A). While we consistently noted a small number of ambiguous-looking fish by external inspection at 3.5, 4 and 6 months of ATR treatment, histological gonadal analysis after six months of treatment confirmed the correct sex assignment for all fish (Fig 2B). Despite previous reports that ATR or estrogen exposure in either a laboratory or field setting resulted in feminized male frogs with testicular oogenesis [10], we found no obvious ovi-testes phenotype in ATR exposed fish. This difference may reflect the length of ATR exposure or the older age of fish examined in our study. Similar to our findings, chronic exposure to estrogens also led to an increase in the ratio of female fish, but showed no intersex fish [26]. At this time we are unable to eliminate the possibility that these effects reflect the actions of an active ATR metabolite, although others showed previously that ATR persists in similar aqueous conditions at doses of 5 µg/L [16]. Nonetheless, our results show that exposure to environmentally relevant but high doses of ATR dramatically increased aromatase levels and increased the percentage of female fish.

### ATR activates all NR5A receptors

**Figure 3:**
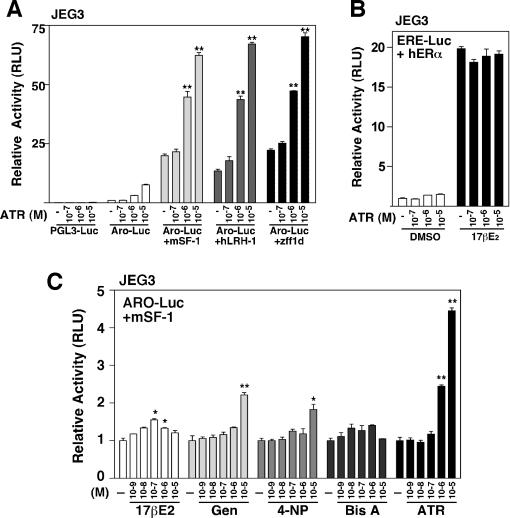
ATR activates NR5A receptors, but does not activate ERα. A. Luciferase activity is shown after transfection of human placental JEG3 cells with ARO-Luc reporter (200 ng) and the parent reporter (pGL3-Luc) with mSF-1, hLRH-1 or zff1d expression vectors (25 ng). Drug treatments with EDCs at the dose indicated was for 24 hrs. B. JEG3 were transfected with the ERE-Luc (50 ng) and hERα (5 ng) and treated with 17βE2 (0.1 µM) with or without ATR (0.1–10 µM). Cells were treated with drug for 24 hr. C. Luciferase activities in JEG3 cells following treatment with EDCs, concentrations are indicated (ranging from 1 nM to 10 µM). All cells were transfected with mSF-1 and Aro-Luc with concentrations as described for panel A. T-test analysis reveal statistical significance with **p<0.01, *p<0.05.

To determine if ATR might directly affect NR5A receptor activity, and thus account for the increased levels of endogenous zcyp19a1 transcripts, cellular reporter assays were performed. Using JEG3 human placental cells, which contain modest amounts of SF-1, ATR treatment activated the aromatase promoter in a dose-dependent manner (Fig 3A). Similar activation was observed with other SF-1 reporters including those with isolated NR5A binding sites (Fig S1A). However no activation was observed using the parent reporter or with mutant SF-1 binding sites (Fig 3A and S1B) showing that ATR effects depend on DNA binding and receptor occupancy of the promoter, as suggested previously [35], [38]. Activation by ATR was enhanced greatly by overexpression of mSF-1 in JEG3 or HepG2 cells (Fig 3A and 5A), similar to results obtained in human H295R adrenal cortical cells [38]. However, while the response in H295R cells was shown to be selective for SF-1 [35], we find that in all responsive cell lines ATR can also activate hLRH-1 (NR5A2) and its close zebrafish ortholog ff1d, which is expressed in embryonic male gonads [24](Fig 3A). Moreover, knocking-down hLRH-1 in human HepG2 liver cells greatly attenuated ATR activation of the aromatase reporter demonstrating a dependence on this NR5A receptor (Fig S5). Based on these findings, we suggest that all NR5A subfamily receptors will be responsive to ATR.

**Figure 4:**
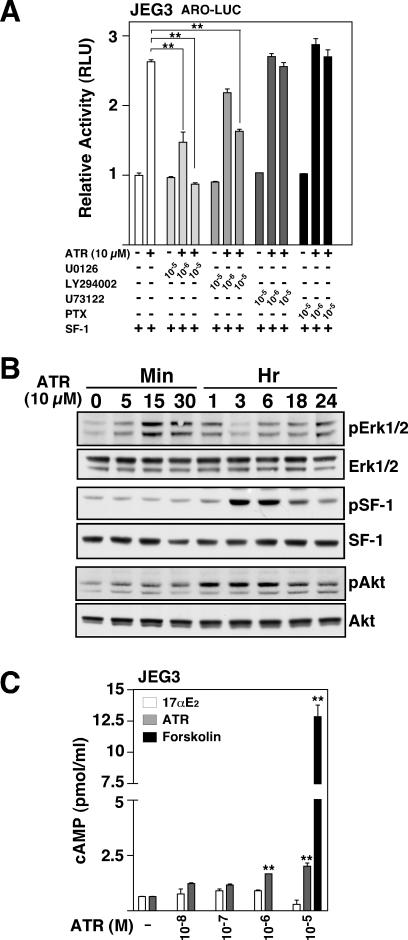
ATR activates the MAPK, PI3K and modestly stimulates cAMP production. A. Pharmacological inhibitors of MAPK (U0126), PI3K (LY294002) or Gi (PTX), and PLC (U73122) signaling were added, with ATR (10 µM) or without (DMSO), and with mSF-1 (25 ng), as indicated (+). All inhibitors were added 60 min prior to ATR treatment. B. Western blotting was performed using antibodies against phospho-ERK1/2, total ERK, phospho-SF-1, Flag, phospho-AKT and total AKT using JEG3 cellular extracts transfected with Flag-tagged mSF-1 (1 µg). Twenty-four hours after transfection, cells were starved for 3 hrs and treated with 10 µM of ATR for the indicated times (5 min to 24 hrs). C. Levels of total cellular cAMP (pmol/ml) were determined in lysed JEG3 cellular extracts according to Materials and Methods. Cells were treated for 30 min with increasing concentrations of drug (estradiol, ATR, or forskolin) as indicated (10 nM to 10 µM) following serum starvation for 24 hrs as described in Materials and Methods. T-test analysis reveal statistical significance with **p<0.01, *p<0.05.

**Figure 5:**
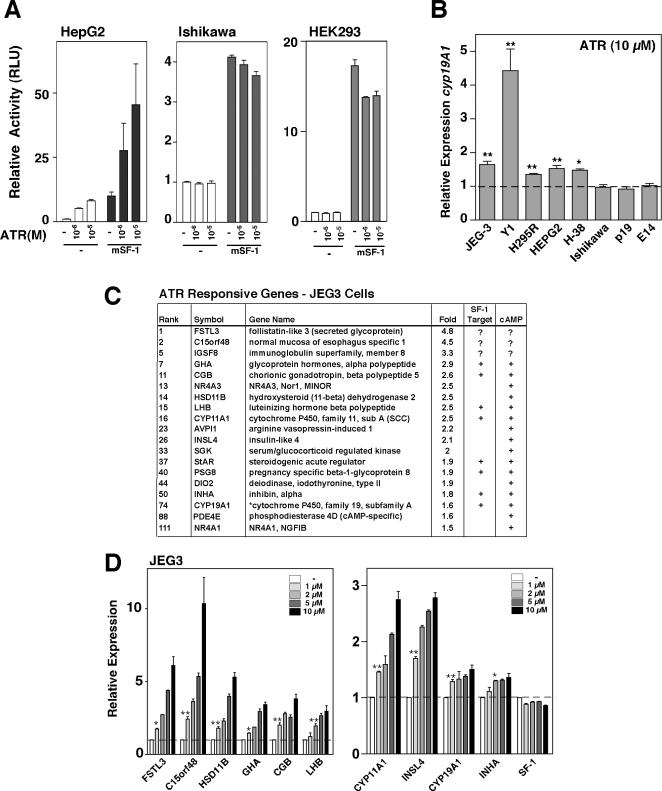
ATR is cell-specific and induces a cluster of genes involved in hormonal responses. A. Relative luciferase activities for the ARO-Luc reporter are shown in responsive (HepG2, human liver) and non-responsive (Ishikawa, uterus, HEK293, embryonic kidney) cell lines following transfection of mSF-1 (25 ng) with increasing doses of ATR. B. Relative levels of endogenous Cyp19A1 are shown for different cell lines after ATR treatment (10 µM, 24 hrs). Cell lines that showed a statistically significant increase in Cyp19A1 expression when compared to DMSO treatment indicated. C. List of endocrine/reproductive related genes with their relative rank order as defined by their fold enrichment following ATR treatment. Known SF-1 target genes and those that are cAMP responsive are indicated as (+). D. Relative expression levels of transcripts in JEG3 cells (without transfection of SF-1) are shown after DMSO (-) or treatment with ATR (1 to 10 µM). JEG3 cells were treated with the indicated doses of ATR for 24 hrs and RT-qPCR analysis was carried out using validated primers as indicated in Table S2. T-test analysis reveal statistical significance with **p<0.01, *p<0.05.

In contrast to the activation of the mammalian and fish aromatase reporter, ATR failed to activate an ERE-reporter in the presence of hERα and estradiol (Fig 3B), consistent with ATR inability to activate the estrogen responsive zcyp19a2 in vivo (this study and [31]). Furthermore, no significant activation of NR5A was observed with other estrogenic compounds, although a reproducible but modest increase in reporter activity was noted with genistein (Fig 3C). Thus, out of the major classes of EDCs, NR5A receptors appear to be selectively and highly responsive to ATR.

Further studies aimed at discerning the cellular mechanisms of ATR revealed that inhibitors of both the MAPK and PI3K decreased or eliminated activation by ATR, whereas disrupting Gi, some Gq (PTX), as well as PLC signaling did not block the effects of ATR (Fig 4A). These results were consistent with ATR's rapid activation of the MAPK pathway, followed by peak phosphorylation of SF-1, and activation of the PI3K pathway as determined by phosphorylation of Akt/PKB (Fig 4B). Furthermore, because others have suggested that ATR inhibits phosphodiesterases and elevates cAMP [9], [39], we asked if ATR directly increased cellular levels of cAMP in JEG3 responsive cells. At activating ATR concentrations (1 µM) we observed a significant and consistent increase in cellular cAMP, albeit at levels much lower than those obtained with forskolin (Fig 4C). Despite these lower levels of cAMP stimulation, forskolin (10 µM), as well as EGF (50 µg/L), activate the aromatase promoter to similar levels observed for ATR (Fig S2). We also asked if ATR might bind directly to SF-1 to either increase DNA binding or act as a ligand. However, ATR neither enhanced nor repressed DNA binding activity to a high affinity SF-1 binding site (Fig S3B). ATR also failed to significantly activate a Gal4-SF-1 ligand binding domain (LBD) fusion constructs at high doses (Fig S3A). Finally, ATR was unable to exchange for, or displace the bound bacterial phospholipid ligand present in the LBD (H.A.I, unpublished data). Collectively, these results suggest that ATR may not directly interact with NR5A receptors, but instead activates three signaling pathways known to activate NR5A receptors, including phosphorylation of SF-1, generation of SF-1 ligands, and increased production of cAMP. All of these effects would be predicted to activate SF-1 further [40], [41], [42], [43].

### ATR increases a cluster of genes involved in endocrine signaling in specific cell types

ATR stimulation of reporter activity (Fig 5A) or induction of endogenous hCyp19A (Fig 5B) were cell-specific with responsive cell lines largely restricted to endocrine-like cells, including adrenal cell lines Y1 and H295R, the human liver cell line HepG2, and primary endometriotic H38 cells; these latter two cell lines express moderate to high levels of SF-1 or LRH-1 [44], [45]. Non-responsive cell lines such as Ishikawa and HEK293 failed to show an ATR response even after overexpression of SF-1 or LRH-1 (Fig 5A, data not shown). The induction of endogenous Cyp19A1 in specific cell lines is consistent with a previous report showing that 10 µM ATR induced aromatase expression and activity in JEG3 and H295R cells, but not in the breast cancer cell line MCF7 [46]. Interestingly, pluripotent cells such as P19 and mouse E14 embryonic stem cells were not responsive to ATR (Fig 5B) despite expression of either SF-1 or LRH-1 (H.A.I. unpublished result); again demonstrating that the ATR response is not restored by the simple addition of exogenous NR5A receptors.

Having surveyed cell lines for their ATR responsiveness, gene profiling was carried out with a human microarray (HEEBO chip) to determine which transcripts in human JEG3 cells are most responsive to ATR (Fig 5C). ATR-responsive genes were determined with or without overexpression of SF-1. Remarkably, a cluster of genes involved in hormone signaling was found to be greatly upregulated following ATR treatment (24hrs, 10 µM). Two of these are rate-limiting for steroid biosynthesis and include steroidogenic acute regulatory protein (StAR) and P450 side-chain cleavage (Cyp11A1), while other responsive genes include known reproductive peptide hormones (INHα, LRH, GSUα, hCGß). Among the top genes, several are known to be SF-1 targets and inspection of other highly induced genes on our list revealed the presence of NR5A binding sites in their promoter regions (FSTL3, c15orf48, Insl4). Further analyses of these top genes showed that their dose-dependent response to ATR treatment (Fig 5D) closely matched the rank order found in the microarray analysis, with FSTL3 and c15orf48 showing the highest response to ATR. Other clusters of ATR-responsive genes relate to metabolism and growth control (Table S1). Levels of SF-1 or LRH-1 transcripts were unchanged by ATR (Fig 5D and data not shown).

**Figure 6:**
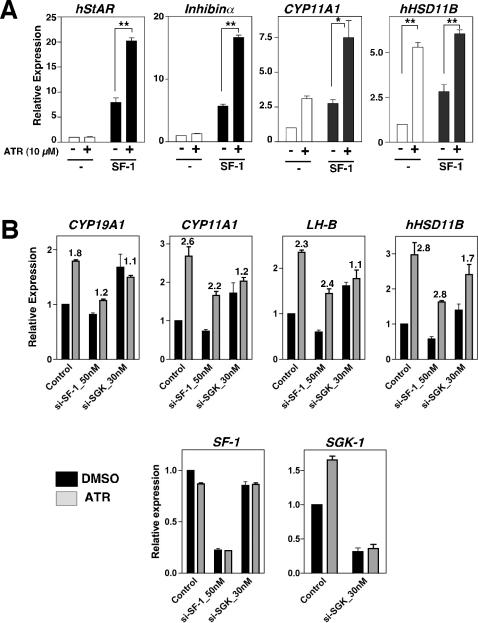
Overexpression of SF-1 enhances ATR effects, while knock-down of SF-1 and SGK-1 diminish or attenuate ATR effects on selective genes. A. Relative endogenous transcript levels in JEG3 cells are shown with and without mSF-1 and with or without ATR treatment (24 hrs, 10 µM). B. Relative expression of transcripts in human JEG3 cells after transfection with si-RNAs directed to human SF-1 (si-SF-1, 50 nM) and human SGK-1 (si-Sgk1, 30 nM) in JEG3 cells, with DMSO (black bars) or with ATR treatment (gray bars, as described above). The fold induction with ATR treatment is indicated above bars. Levels of endogenous human SF-1 and human SGK-1 are shown after si-RNA treatment.

The response to overexpressing SF-1 varied among these endocrine ATR-responsive genes. Some ATR targets increased further after adding SF-1, while others were either slightly repressed or not affected by additional SF-1 (Fig 6A and Fig S4). For known SF-1 downstream targets, such as Cyp19A1, the stimulatory effects of ATR were diminished after knocking-down SF-1 by siRNA (Fig 6B), similar to the loss of the ATR-response observed in HepG2 cells after siRNA knock-down of LRH-1 (Fig S5). Other genes continued to show a significant response to ATR with the caveat that levels of SF-1 persisted at 20% compared to control treated cells.

Nearly all ATR-induced genes identified here are known to be sensitive to cAMP signaling (Fig 5C); the top three responsive genes remain uncharacterized. Surprisingly, one of these ATR targets is the serum glucocorticoid regulated kinase 1 (SGK-1). This tyrosine intracellular kinase has historically been characterized as a glucocorticoid induced gene and regulates sodium channels in the kidney. However SGK-1 is also expressed in endocrine tissues and is thought to mediate FSH peptide hormone signaling in the ovary [47], [48], [49]. Moreover, SGK-1 itself, is upregulated by cAMP. Using siRNA to SGK, we found that reducing cellular SGK-1 also attenuated (HSD11B) or eliminated (Cyp19A1, Cyp11A1, LHß) further enhancement by ATR (Fig 6B). These unexpected results suggest that the intracellular kinase SGK-1, in addition to NR5A receptors, might participate in ATR signaling.

## Discussion

Our in vivo and in vitro analyses of ATR strongly suggest that this widely used herbicide affects hormone signaling and endocrine transcriptional networks in fish and in mammalian cells. Indeed, we found that acute and chronic exposure to ATR significantly increased the endogenous levels of zcyp19a1 encoding gonadal aromatase and altered the normal sex ratio in environmental conditions in a relevant vertebrate model system. Moreover, our cellular data illustrate that ATR induces a cluster of endocrine-related genes, including Cyp19A1. Endocrine-related cell types with a capacity for steroidogenesis appear to be especially sensitive to ATR, as demonstrated by the exquisite cellular specificity of the ATR response. Finally, based on the fact that many of these ATR responsive endocrine targets are downstream of both SF-1 and cAMP signaling, we propose that the selective effects of ATR in endocrine cell types are mediated by convergent regulation of NR5A receptors and elevated cAMP.

Strong field and laboratory evidence show that low levels of atrazine (0.1–3 ppb or µg/L) result in gonadal abnormalities [11] and increase parasitic infections [50], [51], [52]. In contrast to unexposed wilderness areas where no detectable ATR can be found, ATR and its active metabolites easily reach concentrations of 6–12 ppb [11], [53] in shallow ground water near agricultural fields with some heavily exposed agricultural lands cited to reach just under 500 ppb [50]. While the precise environmental levels of ATR can vary greatly, the doses used in our zebrafish study (0.01 to 0.1 µM or 2.1 to 22 ppb) fall within lower levels of ATR contamination, with our lowest dose just under the acceptable amounts of 3 ppb allowed in human drinking water (http://www.epa.gov/safewater/dwh/c-soc/atrazine.html). Based on our data, we would predict that relatively low doses of ATR might adversely influence normal hormone signaling in young fish.

The precise mechanism of action of ATR remains controversial. Consistent with the fact that ATR does not directly interface with classic estrogen signaling [9], ATR had no effect on the estrogen responsive zcyp19a2 or an ERE-Luc reporter. While ATR is suggested to inhibit phosphodiesterase and increases cellular pools of cAMP; others have suggested that PKA signaling is not essential for stimulation of aromatase in H295 cells [35]. However, in JEG3 cells, we do find that ATR easily activates a CRE-LUC reporter (Fig S2B), and can elevate cellular cAMP, albeit at much lower levels than observed for forskolin. Interestingly, these same equivalent concentrations of ATR (10 µM) elicit a prominent effect on aromatase reporter activation, similar to activation observed by either EGF (50 ng/l) or forskolin (1 µM, Fig S2A). Interestingly, we also find that ATR induces the cAMP-phosphodiesterase (PDE) 4D, which is expressed in human fetal tissues [54] and is proposed to mediate inflammatory responses in the myometrium [55]. Our findings are consistent with the known regulation of PDE4D expression by cAMP [55], and suggest that prolonged ATR stimulation partially dampens cAMP signaling; thus the degree of PDE4D might account for low cAMP levels observed after 24 hr treatment with ATR (Fig 4C), and the noted absence of cAMP in other ATR-treated cell lines [35].

The recent suggestion that ATR binds directly to SF-1 [38] would easily account for ATR's cell selectivity that we and others have observed [46]. While such a straightforward mechanism is attractive, our data would argue that the mechanism of ATR is much more complex. Indeed, several lines of evidence suggest that ATR might not function by directly binding to NR5A receptors, at least in the cellular and biochemical assays used in this study. First, introducing or increasing levels of NR5A receptors failed to recapitulate an ATR response in non-responsive cell lines. Second, ATR failed to activate a Gal4-LBD SF-1 fusion or increase DNA binding of SF-1. Third, ATR failed to alter coactivator peptide recruitment using an alpha screen assay (data not shown). Fourth, ATR failed to displace the bacterial phosphatidyl glycerol or an exchanged PIP3 ligand present in the SF-1 ligand binding pocket (H.A.I, unpublished results). Finally, ATR stimulates SF-1 LBD pocket mutants (data not shown), which have been designed to occlude and prevent phospholipid binding in the large 1100 Å^3^ LBD cavity [56]. Taken together, alternative mechanisms must account for this cell specific pharmacological response.

ATR also activates PI3K signaling as evidence by the increase phosphorylation of AKT or protein kinase B. Moreover, inhibitors of PI3K signaling block the ability of ATR to stimulate an SF-1 cellular reporter. Our findings broaden the actions of ATR to other signaling pathways, and are consistent with the fact that SF-1 is responsive to both PKA and PI3K signaling [42], as is the stress induced kinase SGK-1 [57]. Interestingly, SGK-1 is found in many steroidogenic endocrine tissues and is expressed highly in cell lines found to be responsive to ATR, including Y1, JEG3, H295R, H38 endometriotic cells, but not in HEK293 or Ishikawa (data not shown). Although knock-downs of SF-1 [38] and SGK-1 attenuate the ATR response on some target genes, overexpression of SGK-1 and/or SF-1 are insufficient to induce an ATR response in non-responsive cell lines (Fig 5A and data not shown). These data show that while SF-1 and SGK-1 participate in ATR actions, a more comprehensive genome wide survey is needed to identify the full spectrum of ATR targets in the entire endocrine system.

Our profiling of ATR-treated JEG3 cells now provides new ATR-responsive genes for thoroughly studying the potential environmental impact of ATR in wild life and in humans. For example, SGK-1 is of interest given its noted upregulation in the endometrium of women with unexplained infertility suggesting that this kinase is important for implantation and maintenance of early pregnancy [58]. Another gene implicated in human placental health is the top ATR-induced gene, follistatin-like 3 (FSTL3). Elevated FSTL3 transcripts in humans have been linked to hypoxia [59] and intrauterine growth restriction [60], and in mice overexpression of FSTL3 results in gonadal defects [61]. Taken together these findings suggest that further research is needed to determine whether high and/or chronic exposure to ATR in humans compromise normal fertility and contribute to reproductive diseases.

In addition to known endocrine transcripts, it was surprising to find that ATR also induced expression and weakly activated the early response NR4A receptors (Nor1 and NGFI-B, Fig 5 and S2C). It is worth noting that these nuclear receptors promote gluconeogenesis in vivo, and are directly regulated by cAMP in HepG2 liver cells [62]. Nor1 is also reported to directly regulate SGK-1 [63]. In addition to glucose homeostasis, members of the NR4A family are activated in macrophages as part of the inflammatory response and are implicated in TCR-mediated cell death and thymocyte-negative selection [64], [65]. Thus, it is feasible that activation of Nor1 and NGF-IB might account for other physiological effects noted for ATR including decreased survivorship in amphibians [52], [66], and impaired immune responses in rodents [67], [68], [69].

Although our in vivo analysis focused solely on Cyp19A1 expression and sex ratios in exposed zebrafish, the fact that ATR upregulates several peptide hormones and steroidogenic genes in mammalian cells suggests that the in vivo effects of these triazine herbicides will be much broader, extending well beyond estrogen metabolism. Further studies using model organisms, where genomic approaches are feasible, should help to determine the full extent of ATR effects on endocrine signaling and other physiological responses, including the immune response and early embryonic development [70]. Given the current pervasive use and persistence of ATR in the environment, our findings support environmental concerns that ATR poses a potential risk to the reproductive health of young fish and other wild life. We also suggest that further research is needed to determine how this non-estrogenic EDC influences the mammalian embryonic and adult endocrine system.

## Materials and Methods

### Biological Reagents

Antibodies, chemicals, oligonucleotides, plasmids and cell lines used are specified in Text S1.

### Plasmids

Full length mouse SF-1 (mSF-1) was PCR amplified from HA-mSF-1/pCIneo. Full length human LRH-1 (hLRH-1, cloned from human ductal carcinoma T47-D cDNA) was PCR amplified and subcloned into 3x FLAG pcDNA3. Full length zebrafish nr5a ff1d was isolated from zebrafish cDNA obtained from total body RNA by PCR amplification, and subcloned into 3x FLAG pcDNA3. Aromatase-Luc (ARO-Luc) was described previously [56]. ERα and ERE-TATA-Luc vectors were generous gifts from Dr. S Kato (University of Tokyo, Japan).

### Cell Culture and Cell Lines

JEG3, HepG2 and H295R cells were maintained in DMEM/H-21, 4.5g/L glucose supplemented with 10% fetal bovine serum (FBS) and 1x penicillin/streptomycin (P/S). Y1 and human H-38 endometriotic cells were maintained in DMEM/F12 with 15% Horse serum, 2.5% FBS and P/S, MA10 cells were maintained in Waymouth's with 15% horse serum and P/S, p19 cells were maintained in αMEM with 10% FBS, E14 were maintained in DME/H-21 with 15% fetal calf serum and P/S.

For transient transfections, cells were plated at a density of 20,000 cells/well in 24-well plates in phenol red-free DME H-21, 4.5 g/L glucose with 4% charcoal-dextran-stripped (CDS) FBS. Cells were co-transfected with 200 ng ARO-Luc reporter plasmid and 3x FLAG-mSF-1 expression vectors using FuGENE 6 (Roche, Indianapolis, IN). Cells were treated with indicated drugs for 6 hr before harvesting. Thereafter, luciferase activity was determined using the Ventas Microplate Luminometer (Turner Biosystems, Sunnyvale, CA). All transfections were performed in triplicate and repeated at least three times.

Western blot analysis was carried out in JEG3 cells transfected (24 hr) with 3x FLAG-mSF-1 and serum starved in phenol red-free medium. Following serum starvation, cells were treated with drugs for various times as indicated, washed with ice cold phosphate buffered saline (PBS) and lysed with ice cold 20 mM Tris-HCl pH 7.4, 150 mM NaCl, 1 mM EDTA, 1 mM EGTA, 1% NaDeoxycholate, 1% Triton X-100, 0.1% SDS, Complete Protease Inhibitor Cocktail (Roche), 1 mM phenylmethylsulphonyl fluoride, 1x Phosphatase Inhibitor Cocktail I and II (Sigma-Aldrich, St. Louis, MO). Protein concentration was determined using Pierce Coomassie Protein Assay Kit (Pierce, Rockford, IL). Twenty five micrograms of total protein was electrophoresed on NuPAGE 4%–12% Bis-Tris gels (Invitrogen) and separated proteins transferred and probed with antibodies against phospho-Akt (Ser473) (1∶1,000), Akt (1∶1,000), phospho-p44/p42 (1∶1,000), p44/p42 (1∶1,000), pSF-1 (1∶2,500) and Flag M2 (1∶5000). Signal was detected using SuperSignal West Femto Maximum Sensitivity Substrate (Pierce).

Levels of cellular cAMP were determined on whole cell extracts prepared by the protocol provided with the R&D Systems ELISA cAMP Parameter Assay Kit (Minneapolis, MN). All values were normalized to internal standards with a standard curve ranging from 0.78 to 200 pmol/mL, and were measured on a Thermo Malti-Skan Ex plate reader at 450 nm. Two independent assays were carried out with each experimental condition done in triplicate.

### Zebrafish and EDCs exposure

Adult zebrafish (*Danio rerio*) were maintained in recirculating aquarium tank at 28 C on a 14h:10h light:dark cycle. Fertilized eggs were harvested and juveniles were raised until 17 days post fertilization (dpf). Fifteen zebrafish at 17dpf were exposed together in the glass beaker containing 500 mL of aquarium water for 3 days with either DMSO vehicle (0.1%, v/v) as the control group or with multiple classes of EDCs. Total RNAs from 5 fish were pooled and purified using TRIzol (Invitrogen, CA). For assessing ATR effects on the percentage of male and female zebrafish, 17dpf zebrafish was exposed for 6 months to either DMSO, or 10^−7^ M, 10^−6^ M and 10^−5^ M ATR. ATR was diluted from a stock solution and added to fish water; tanks were changed three times per week with fresh ATR. All research involving zebrafish were approved and carried out according to guidelines of the UCSF IACUC committee.

For gonadal histology, fish were anesthetized with 100 mg/L tricaine methanesulphonate (Sigma) and fixed with 95% of EtOH, 4% of formalin and 2% of glacial acetic acid, sectioned (4 µm), and stained with H & E.

### RNA Analysis

Total RNA from cultured cells was extracted using TRIzol. For quantitative PCR (qPCR), cDNA was synthesized from 2 µg of total RNA with Superscript III Reverse Transcriptase (Invitrogen) using random hexamer primers (Amersham Biosciences). RT-qPCR was performed using the SYBR Green PCR Master Mix kit (Applied Biosystems, Foster City, CA) with gene-specific primer pairs. All primer sets were designed by Primer Express v2.0; Applied Biosystems and validated to ensure amplification of a single product with appropriate efficiency. Data obtained from the PCR reaction was analyzed using the comparative C_T_ method (User Bulletin No. 2, PerkinElmer Life Sciences). All primer sequences are listed in Table S2.

### Microarray Analysis

HEEBO (Human Exonic Evidence Based Open-source) arrays were printed in-house at the Center for Advanced Technology (UCSF, CA). Total RNAs from JEG3 cells were prepared using RNeasy (Qiagen, Valencia, CA). For each array, total RNA (12.5 µg) obtained for each sample was reverse transcribed and labeled with amino-allyl dUTP using reverse transcriptase III and Oligo-dT/random primer mixture (Invitrogen, Carlsbad, CA). cDNA was purified using MinElute PCR Purification Kit (Qiagen, CA). Two µg of cDNA was coupled to Cy3 or Cy5 dye (Monofunctional NHS-ester Dye, Amersham, Piscataway, NJ). Cy3/Cy5 labeled cDNAs were purified, combined, and hybridized in a sealed chamber at 65 C for 48 hrs. Slides were washed in Solution I (3x SSC, 0.2% SDS) at 55C 1 min, Solution II (1x SSC) 1min twice at room temperature (RT), Solution III (0.2x SSC) 1 min at RT and quickly dried by centrifugation. Hybridized slides were scanned using Axon Slide Scanner 4000B and data were analyzed by Genepix 6.0 software (Molecular Devices, CA).

## Supporting Information

Text S1 (0.02 MB DOC)

Figure S1 A. Luciferase Activity is shown for different reporters, as indicated above bar graphs with or without SF-1 and with increasing concentrations of ATR added. B. Luciferase activity is shown with a hypothalamic SF-1 target gene using a wild type and mutant promoter. The SF-1 binding site is mutated in the mutant promoter (mutant SF-1 RE). The promoter is ataxin 2 binding protein.
(0.11 MB PDF)

Figure S2 A. Luciferase activity in JEG-3 cells following treatment with ATR (10 µM), EGF (50 µg/L) or Forskolin (10 µM) for 24 hrs. All cells were transfected with 25 ng of mSF-1 and 200 ng of Aro-Luc reporter plasmid. B. Luciferase activity for JEG-3 cells transfected with the pCRE-Luc (200 ng, pCRE-Luc, Clontech) and with indicated amount of ATR added for 24 hrs. C. JEG-3 cells were transfected with 5x OH-Luc reporter (200 ng) and NGFIB (10 ng), treated with indicated amount of ATR added for 24 hrs.
(0.14 MB PDF)

Figure S3 A. GAL-4 fused luciferase activity is shown using 200 µg of GAL-4 reporter, pFR-Luc (Stratagene), as indicated with 100 µg of mSF-1 (hinge-LBD aa105 to 462) or hLRH-1 (aa198 to 562), with increasing concentrations of ATR added. B. EMSA assay. For EMSA binding assays, the mMIS SF-1 binding site was used. Sequences are described in Text S1. The concentration of ATR is indicated, and was added to binding buffer and then incubated with purified mSF-1 containing the entire DNA binding domain.
(0.22 MB PDF)

Figure S4 A. Cluster analysis of significant (P<0.001) changes in gene expression after ATR (10 µM, 24 hrs) compared to DMSO, and with or without transfection of mouse SF-1 (SF-1+ or SF-1-). The relative fold change is indicated on the bar legend to the left. B. Relative expression levels in JEG3 cells (with/ without transfection of mSF-1) after DMSO (-) or treatment with ATR (10 µM). JEG3 cells were transfected with 5 µg of mSF-1 and treated with the ATR for 24hrs with RT-qPCR analysis carried out using validated primers as indicated in Table S2. Endogenous expression levels of hSF-1 are shown by RT-qPCR (right upper panel).
(0.19 MB PDF)

Figure S5 HepG2 liver cells were transfected with 200 ng of ARO-Luc and with either 100 ng siRNA of control (sicRNA) or hLRH-1 (si-hLRH-1), left panel. Relative luciferase activities after drug treatment are shown as mean values +/−s. d. Endogenous transcript levels of hLRH-1 are shown as determined by RT-qPCR, right panel.
(0.09 MB PDF)

Table S1 (0.05 MB PDF)

Table S2 (0.06 MB DOC)
