# Supplementary material for: The Herbicide Atrazine Activates Endocrine Gene Networks via Non-Steroidal NR5A Nuclear Receptors in Fish and Mammalian Cells
Source: PLoS One. 2008 May 7;3(5):e2117. doi: 10.1371/journal.pone.0002117 (PMC2362696; doi:10.1371/journal.pone.0002117)
Supplement: Text S1 — (0.02 MB DOC) [file pone.0002117.s001.doc]

# A list of Antibodies, Chemicals and Oligos used for mutagenesis and EMSA is provided.

# 1. Antibodies

Phospho-Akt antibody; Cell Signaling Technology

Akt antibody; Cell Signaling Technology

Phospho-p44/42 MAP kinase antibody; Cell Signaling Technology

p44/42 MAP kinase antibody; Cell Signaling Technology, Danvers, MA

Phospho SF-1 antibody; created by Ingraham lab, UCSF

Flag M2 antibody; Sigma-Aldrich, St. Louis, MO

### 2. Chemicals

Atrazine; Sigma-Aldrich

17α-Estradiol; Sigma-Aldrich

17β-Estradiol; Sigma-Aldrich

Genistein; TOCRIS bioscience, Glasgow, UK

Bis-phenol A; TOCRIS

4-Nonylphenol; TOCRIS

DMSO; Pierce, Rockford, IL

LY294002; TOCRIS

U73122; TOCRIS

PTX; TOCRIS

U0126 (MEK1/2 inhibitor); Cell Signaling Technology

Recombinant human EGF; R&D systems, Inc. Minneapolis, MN

Forskolin; TOCRIS

**3. Mutagenesis Oligomers**

**Name Sequence**

***Sense*** ***Antisense***

mSF-1_S203A CCAGAGCCCTATGCCAGCCCCCCA AGGGGGGCTGGCATAGGGCTCTGG

mSF-1_A270W GCAGAATGTGGGACCAGACC GGTCTGGTCCCACATTCTGC

mSF-1_L345F GCTGGCTCCCTGTTCCACAGCCTGGTG CACCAGGCTGTGGAACAGGGAGCCAGC

mSF-1_A454L CTGAGCATGCAGCTGAAGGAGTACCTG CAGGTACTCCTTCAGCTGCATGCTCAG

**4. EMSA Oligomers**

mMIS wt sense

5' -GCCAGGCACTGTCCC**CCAAGGTCA**CCTTTGGTGTTGATA-3'

mMIS wt antisense

5' -TATCAACACCAAAGG**TGACCTTGG**GGGACAGTGCCTGGC-3'

**Bold** = SF-1 binding site
