# Supplementary material for: The Herbicide Atrazine Activates Endocrine Gene Networks via Non-Steroidal NR5A Nuclear Receptors in Fish and Mammalian Cells
Source: PLoS One. 2008 May 7;3(5):e2117. doi: 10.1371/journal.pone.0002117 (PMC2362696; doi:10.1371/journal.pone.0002117)
Supplement: Figure S1 — A. Luciferase Activity is shown for different reporters, as indicated above bar graphs with or without SF-1 and with increasing concentrations of ATR added. B. Luciferase activity is shown with a hypothalamic SF-1 target gene using a wild type and mutant promoter. The SF-1 binding site is mutated in the mutant promoter (mutant SF-1 RE). The promoter is ataxin 2 binding protein. (0.11 MB PDF) [file pone.0002117.s002.pdf]

Supplemental Figure 1

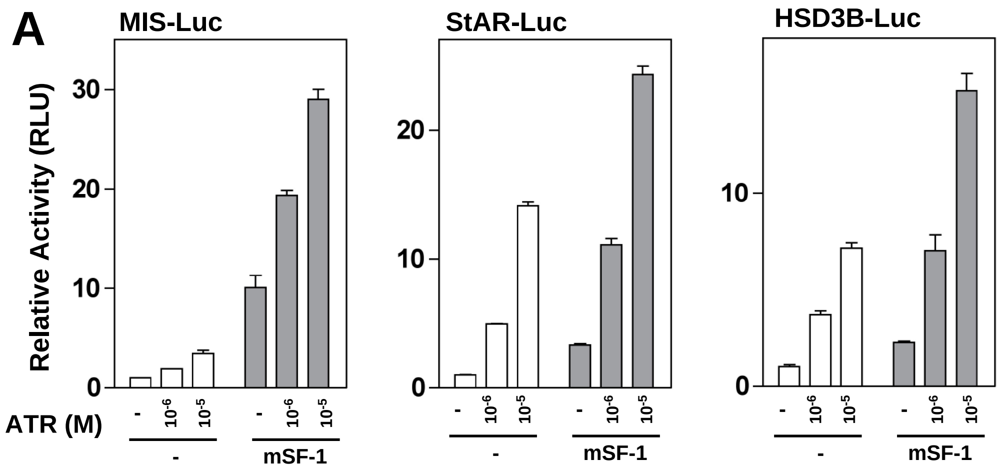

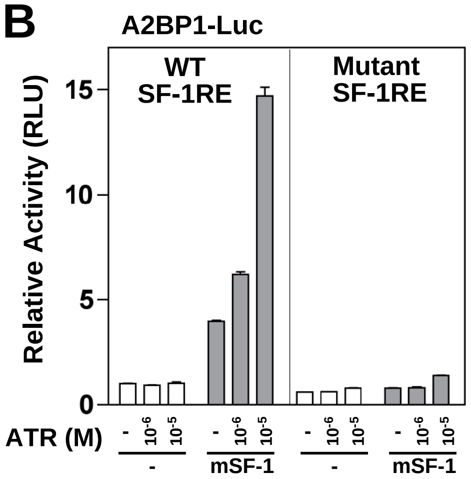

A. Luciferase Activity is shown for different reporters, as indicated above bar graphs with or without SF-1 and with increasing concentrations of ATR added.

B. Luciferase activity is shown with a hypothalamic SF-1 target gene using a wild type and mutant promoter. The SF-1 binding site is mutated in the mutant promoter (mutant SF-1 RE). The promoter is ataxin 2 binding protein.
