# Supplementary material for: The Herbicide Atrazine Activates Endocrine Gene Networks via Non-Steroidal NR5A Nuclear Receptors in Fish and Mammalian Cells
Source: PLoS One. 2008 May 7;3(5):e2117. doi: 10.1371/journal.pone.0002117 (PMC2362696; doi:10.1371/journal.pone.0002117)
Supplement: Figure S2 — A. Luciferase activity in JEG-3 cells following treatment with ATR (10 µM), EGF (50 µg/L) or Forskolin (10 µM) for 24 hrs. All cells were transfected with 25 ng of mSF-1 and 200 ng of Aro-Luc reporter plasmid. B. Luciferase activity for JEG-3 cells transfected with the pCRE-Luc (200 ng, pCRE-Luc, Clontech) and with indicated amount of ATR added for 24 hrs. C. JEG-3 cells were transfected with 5x OH-Luc reporter (200 ng) and NGFIB (10 ng), treated with indicated amount of ATR added for 24 hrs. (0.14 MB PDF) [file pone.0002117.s003.pdf]

Supplemental Figure 2

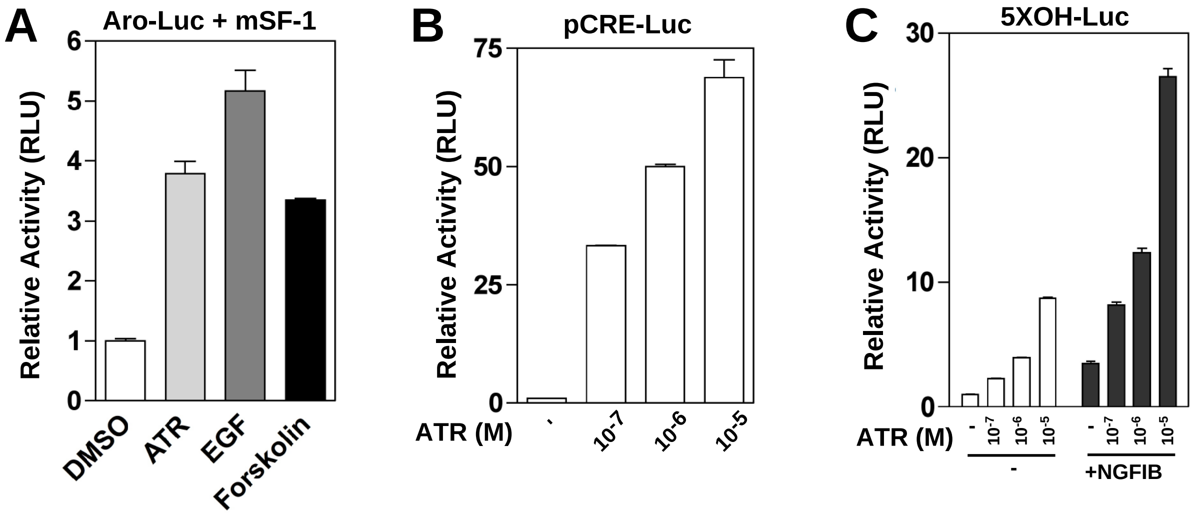

A. Luciferase activity in JEG-3 cells following treatment with ATR (10 μM), EGF (50 μg/L) or Forskolin (10 μM) for 24 hrs. All cells were transfected with 25 ng of mSF-1 and 200 ng of Aro-Luc reporter plasmid.

B. Luciferase activity for JEG-3 cells transfected with the pCRE-Luc (200 ng, pCRE-Luc, Clontech) and with indicated amount of ATR added for 24 hrs.

C. JEG-3 cells were transfected with 5x OH-Luc reporter (200 ng) and NGFIB (10 ng), treated with indicated amount of ATR added for 24 hrs.
