# Supplementary material for: The Herbicide Atrazine Activates Endocrine Gene Networks via Non-Steroidal NR5A Nuclear Receptors in Fish and Mammalian Cells
Source: PLoS One. 2008 May 7;3(5):e2117. doi: 10.1371/journal.pone.0002117 (PMC2362696; doi:10.1371/journal.pone.0002117)
Supplement: Figure S3 — A. GAL-4 fused luciferase activity is shown using 200 µg of GAL-4 reporter, pFR-Luc (Stratagene), as indicated with 100 µg of mSF-1 (hinge-LBD aa105 to 462) or hLRH-1 (aa198 to 562), with increasing concentrations of ATR added. B. EMSA assay. For EMSA binding assays, the mMIS SF-1 binding site was used. Sequences are described in Text S1. The concentration of ATR is indicated, and was added to binding buffer and then incubated with purified mSF-1 containing the entire DNA binding domain. (0.22 MB PDF) [file pone.0002117.s004.pdf]

Supplemental Figure 3

A

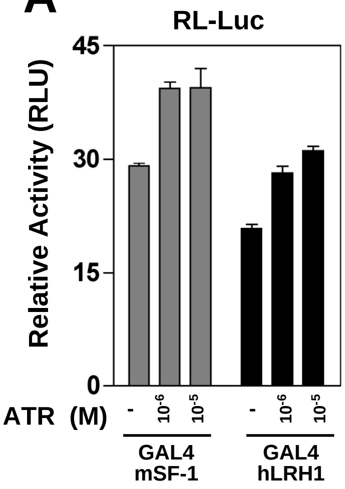

B

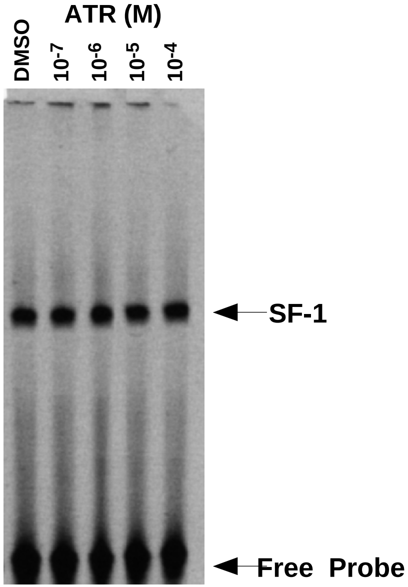

A. GAL-4 fused luciferase activity is shown using 200 µg of GAL-4 reporter, pFR-Luc (Stratagene), as indicated with 100 µg of mSF-1 (hinge-LBD aa105 to 462) or hLRH-1 (aa198 to 562), with increasing concentrations of ATR added.

B. EMSA assay. For EMSA binding assays, the mMIS SF-1 binding site was used. Sequences are described in Supporting Information. The concentration of ATR is indicated, and was added to binding buffer and then incubated with purified mSF-1 containing the entire DNA binding domain.
