# Supplementary material for: The Herbicide Atrazine Activates Endocrine Gene Networks via Non-Steroidal NR5A Nuclear Receptors in Fish and Mammalian Cells
Source: PLoS One. 2008 May 7;3(5):e2117. doi: 10.1371/journal.pone.0002117 (PMC2362696; doi:10.1371/journal.pone.0002117)
Supplement: Figure S4 — A. Cluster analysis of significant (P<0.001) changes in gene expression after ATR (10 µM, 24 hrs) compared to DMSO, and with or without transfection of mouse SF-1 (SF-1+ or SF-1-). The relative fold change is indicated on the bar legend to the left. B. Relative expression levels in JEG3 cells (with/ without transfection of mSF-1) after DMSO (-) or treatment with ATR (10 µM). JEG3 cells were transfected with 5 µg of mSF-1 and treated with the ATR for 24hrs with RT-qPCR analysis carried out using validated primers as indicated in Table S2. Endogenous expression levels of hSF-1 are shown by RT-qPCR (right upper panel). (0.19 MB PDF) [file pone.0002117.s005.pdf]

Supplemental Figure 4

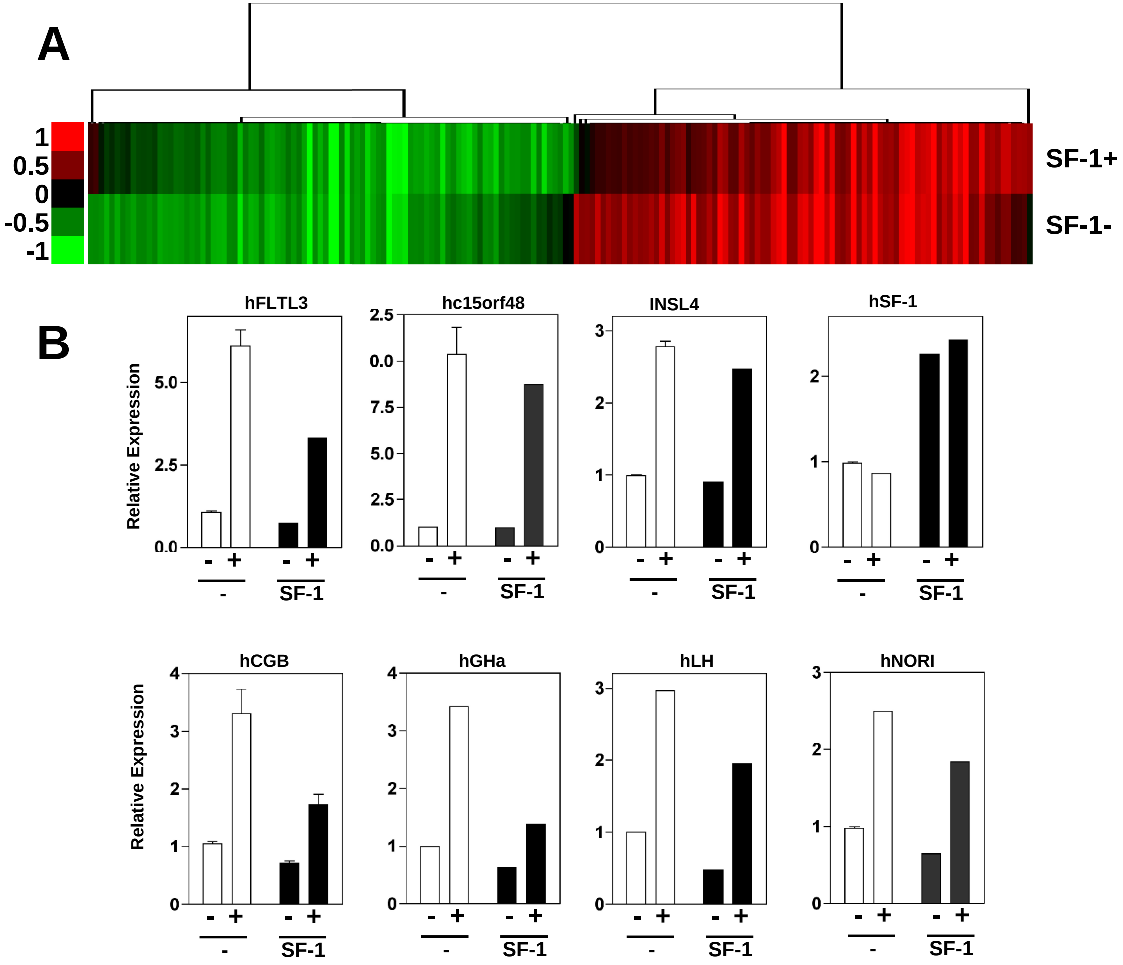

A. Cluster analysis of significant (P<0.001) changes in gene expression after ATR (10 μM, 24 hrs) compared to DMSO, and with or without transfection of mouse SF-1 (SF-1+ or SF-1-). The relative fold change is indicated on the bar legend to the left.

B. Relative expression levels in JEG3 cells (with/ without transfection of mSF-1) after DMSO (-) or treatment with ATR (10 μM). JEG3 cells were transfected with 5 μg of mSF-1 and treated with the ATR for 24hrs with RT-qPCR analysis carried out using validated primers as indicated in Table S2. Endogenous expression levels of hSF-1 are shown by RT-qPCR (right upper panel).
