# Supplementary material for: The Herbicide Atrazine Activates Endocrine Gene Networks via Non-Steroidal NR5A Nuclear Receptors in Fish and Mammalian Cells
Source: PLoS One. 2008 May 7;3(5):e2117. doi: 10.1371/journal.pone.0002117 (PMC2362696; doi:10.1371/journal.pone.0002117)
Supplement: Figure S5 — HepG2 liver cells were transfected with 200 ng of ARO-Luc and with either 100 ng siRNA of control (sicRNA) or hLRH-1 (si-hLRH-1), left panel. Relative luciferase activities after drug treatment are shown as mean values +/−s. d. Endogenous transcript levels of hLRH-1 are shown as determined by RT-qPCR, right panel. (0.09 MB PDF) [file pone.0002117.s006.pdf]

Supplemental Figure 5

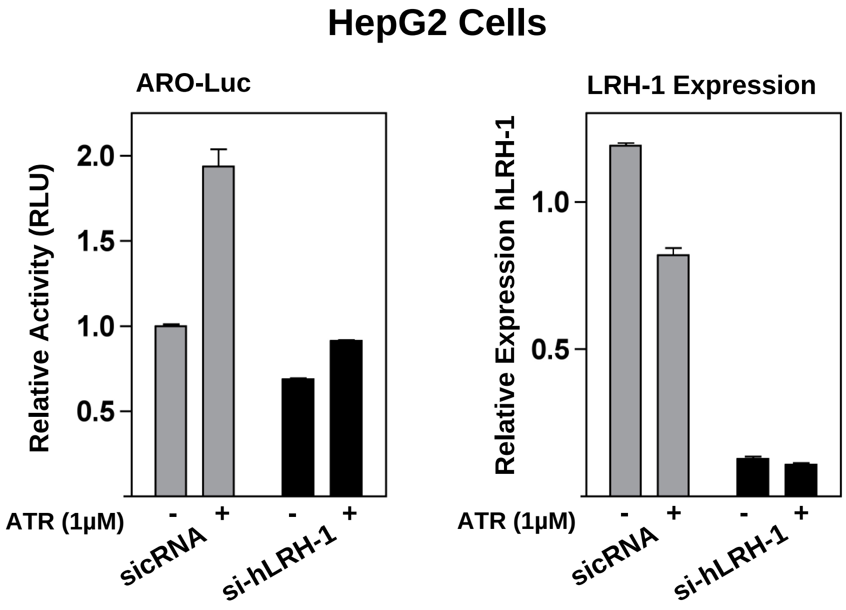

HepG2 liver cells were transfected with 200 ng of ARO-Luc and with either 100 ng siRNA of control (sicRNA) or hLRH-1 (si-hLRH-1), **left panel**. Relative luciferase activities after drug treatment are shown as mean values +/- s. d. Endogenous transcript levels of hLRH-1 are shown as determined by RT-qPCR, **right panel**.
