# Supplementary material for: The Herbicide Atrazine Activates Endocrine Gene Networks via Non-Steroidal NR5A Nuclear Receptors in Fish and Mammalian Cells
Source: PLoS One. 2008 May 7;3(5):e2117. doi: 10.1371/journal.pone.0002117 (PMC2362696; doi:10.1371/journal.pone.0002117)
Supplement: Table S1 — (0.05 MB PDF) [file pone.0002117.s007.pdf]

Supplemental Table 1

Selected list of lipid metabolism and growth control ATR-responsive genes among the top 150 candidates.

Rank	Symbol	Gene Name	Fold
6	WNT10B	wingless-type MMTV integration site family	2.9
18	Plekha2	pleckstrin homology domain containing, family A	2.3
21	LRP8	low density lipoprotein receptor-related protein 8	2.2
20	ACACB	acetyl-Coenzyme A carboxylase beta	2.2
29	ELOVL3	elongation of very long chain fatty acids-like 3	2
44	DIO2	deiodinase, iodothyronine, type II	1.9
136	FADS3	fatty acid desaturase 3	1.4
22	EGFL7	EGF-like-domain, multiple 7	2.2
25	FGFR3	fibroblast growth factor receptor 3	2.1
27	TGFB1	transforming growth factor, beta 1	2.1
30	PGF	placental growth factor	2
54	RHOV	ras homolog gene family	1.8
68	TNFSF10	tumor necrosis factor superfamily	1.7
69	CCNL1	cyclin L1	1.7
71	CREB3L2	cAMP responsive element binding protein	1.6
79	CCND3	cyclin D3	1.6
126	MAPK4	mitogen-activated protein kinase 4	1.4
