# Supplementary material for: The Herbicide Atrazine Activates Endocrine Gene Networks via Non-Steroidal NR5A Nuclear Receptors in Fish and Mammalian Cells
Source: PLoS One. 2008 May 7;3(5):e2117. doi: 10.1371/journal.pone.0002117 (PMC2362696; doi:10.1371/journal.pone.0002117)
Supplement: Table S2 — (0.06 MB DOC) [file pone.0002117.s008.doc]

# Supplemental Table 2 - Primer Sequences for *qPCR*

| **Name** |  | **Accession** | ***Forward (5’-3’)*** | ***Reverse (5’-3’)*** |
| --- | --- | --- | --- | --- |
| hCyclo |  | NM_203431 | TTTCATCTGCACTGCCAAGA | TTGCCAAACACCACATGCT |
| hCyp19A1 |  | NM_031226 | CCCTTCTGCGTCGTGTCA | CTTTCGTCCAAAGGGATCCT |
| hCyp11A1 |  | NM_000781 | TTCTGCCGCTTCCAGAAAT | TTGTACATCGGCCCAAACTT |
| hStAR |  | NM_000349 | CCCATGGAGAGGCTCTATGAA | GTTCCACTCCCCCATTGCT |
| hINHa |  | NM_002191 | GGGAACGGTGGATCGTGTA | CCCACAACCACCATGACAGT |
| zCyp19A1 |  | NM_131154 | CATTGTGCGTGTCTGGATCA | GCTGACGACCTGTTCAAGATG |
| zCyp19A2 |  | NM_131642 | CATGCAGCCAGTAGAAGAGGAT | TGAGCTCGCGGGATGA |
| hFSTL3 |  | NM_005860 | CAAGAGGGCTGGGCATTC | GAGAAAAGCAGTCATGCCTCTTC |
| hC15orf48 |  | NM_032413 | CCTGATGAAAAGGAAGGAACTCA | ACCCGCCGCCACAGT |
| hGHα |  | NM_000735 | TCTCCATTCCGCTCCTGAT | GGTTTTCCTGTAGCGTGCATT |
| hCGβ |  | NM_033142 | CCGAGGTATAAAGCCAGGTACA | TGTCCCGCCCATGCT |
| hLHβ |  | NM_000894 | AGTGTGCATCACCGTCAACA | GCACGCGCATCATGGT |
| hHSD11β |  | NM_005525 | GGGTCAAGGTCAGCATCATC | TGACCCACGTTTCTCACTGACT |
| hINSL4 |  | NM_002195 | GTCCCAAAGAAATGGTGTCAAC | TCTGATGTCGTACCTAAGGCTTGT |
| hSF-1 |  | NM_004959 | TTCTGCCGCTTCCAGAAAT | TTGTACATCGGCCCAAACTT |
| hSGK1 |  | NM_005618 | TTTGAGCGCTAACGTCTTTCTG | AGCCTCAGTTTTCACCGTCAT |
